# Efficient detection of African Swine Fever Virus using minimal equipment through a LAMP PCR method

**DOI:** 10.3389/fcimb.2023.1114772

**Published:** 2023-01-27

**Authors:** Jose Alejandro Bohorquez, Saraswathi Lanka, Rosa Rosell, Marta Pérez-Simó, Mònica Alberch, Fernando Rodriguez, Llilianne Ganges, Carol W. Maddox

**Affiliations:** ^1^ Veterinary Diagnostic Laboratory, College of Veterinary Medicine, University of Illinois at Urbana-Champaign, Urbana, IL, United States; ^2^ WOAH Reference Laboratory for Classical Swine Fever, IRTA-CReSA, Barcelona, Spain; ^3^ Unitat mixta d’Investigació IRTA-UAB en Sanitat Animal, Centre de Recerca en Sanitat Animal (CReSA), Bellaterra, Barcelona, Spain; ^4^ IRTA, Programa de Sanitat Animal, Centre de Recerca en Sanitat Animal (CReSA), Bellaterra, Barcelona, Spain; ^5^ Departament d’Acció Climàtica, Alimentació i Agenda Rural, Generalitat de Catalunya, Barcelona, Spain; ^6^ Department of Pathobiology, College of Veterinary Medicine, University of Illinois at Urbana-Champaign, Urbana, IL, United States

**Keywords:** African swine fever, surveillance, disease control, LAMP PCR, isothermal amplification, point of care testing

## Abstract

African swine fever virus (ASFV) currently represents the biggest threat to the porcine industry worldwide, with high economic impact and severe animal health and welfare concerns. Outbreaks have occurred in Europe and Asia since ASFV was reintroduced into the continent in 2007 and, in 2021, ASFV was detected in the Caribbean, raising alarm about the reemergence of the virus in the Americas. Given the lack of vaccines against ASFV, control of the virus relies on molecular surveillance, which can be delayed due to the need for sample shipment to specialized laboratories. Isothermal PCR techniques, such as LAMP, have become increasingly attractive as point-of-care diagnostic tools given the minimal material expense, equipment, and training required. The present study aimed to develop a LAMP assay for the detection of ASFV. Four LAMP primer sets were designed, based on a consensus sequence for the ASFV p72 gene, and were tested using a synthetic plasmid containing the cloned ASFV p72 target gene as a positive control. Two primer sets, were selected for further validation, given their very short time for amplification. Both primer sets showed thermal stability, amplifying the ASFV DNA at temperatures between 60-70°C and proved to have an analytical limit of detection as low as one ASFV-plasmid DNA copy/µL, using both fluorometric and colorimetric methods. The selected primers did not yield false positive or cross reactive results with other common swine pathogens, showing high specificity. Testing of DNA-spiked samples showed that LAMP amplification was not affected by the nature of the matrices, including oral fluids, tonsils, blood, or rectal swabs. The primer sets were able to detect the two more prevalent ASFV genotypes in the field. Taken together, the results show that ASFV-LAMP-BG2 and ASFV-LAMP-BG3 would be a useful tool for rapid, highly sensitive on-site diagnostic testing.

## 1 Introduction

African swine fever (ASF) currently poses one of the most worrisome threats to the porcine industry worldwide (Costard et al., 2009; Sauter-Louis et al., 2021a; Gladue and Borca, 2022). The disease is characterized by a severe hemorrhagic disease, leading to high lethality. Its complex epidemiology, which involves different transmission cycles taking place in domestic or wild environments, complicates the control of ASF (Tulman et al., 2009; Pietschmann et al., 2016; Ramirez-Medina et al., 2022). Its etiological agent, the ASF virus (ASFV), is the sole member of the Asfivirus genus and the Asfarviridae family (Alonso et al., 2018). Currently, there is no commercially available vaccine that can be used globally in all ASFV affected regions. Therefore, the disease control relies on testing and elimination of affected animals, as well as those within a determined radius surrounding the affected area. Other control measures include movement restrictions of pork-derived products from the affected countries, as well as heightened biosecurity measures in domestic pig farms (Costard et al., 2009; Bosch-Camós et al., 2021). The direct impact of the disease and the control measures associated with it have led to losses as high as $130 billion in some of the affected countries, mostly targeting smallholders (Weaver and Habib, 2020; Brown et al., 2021; Frezal et al., 2021; Nguyen-Thi et al., 2021). Additionally, ASFV outbreaks have indirect repercussions along a wide range of industries, leading to further losses and market compensations that destabilize the economy (Weaver and Habib, 2020; Frezal et al., 2021). Considering this, ASFV is considered a high-risk pathogen, handled under biosafety level 3 (BSL3) conditions and included in the list of mandatory notification to the World Organization for Animal Health (WOAH, formerly OIE) (World organisation for animal health, 2019a).

ASFV is an icosahedral shape virus, measuring ≈ 200 nm in diameter, with a dsDNA genome between 170 – 190 kb long, encoding 150 – 170 open reading frames (Alonso et al., 2018; Wang et al., 2021b). It was discovered in sub-Saharan Africa in the 1920s, where it has remained endemic ever since, and was spread to Europe in the 1960s and to the Americas a decade later (Costard et al., 2013; Schambow et al., 2022). Following eradication in the 1990s, ASFV was reintroduced into the Eurasian continent in 2007 through Georgia and, from there, ASFV has spread both east and west-ward (Rowlands et al., 2008; Costard et al., 2013). This led to a series of outbreaks during the early 2010s, culminating in the introduction of the virus in large pork-producing countries, such as China and Germany (Sauter-Louis et al., 2021b; Ito et al., 2022). Notably, in 2021, ASFV was detected in the Dominican Republic and Haiti, raising alarms about the reintroduction of the disease into the Americas (Gonzales et al., 2021; Stepien and Cole, 2021; Schambow et al., 2022).

Therefore, efficient ASFV surveillance, based on serologic or molecular tests, has become one of the most important tools for avoiding the introduction of the disease into a country or taking the necessary actions, once it has been detected. However, the high degree of virulence of the pandemic ASFV strain causes rapid death in most infected animals (Borca et al., 2020; Gallardo et al., 2021) and some of them die before they can generate an effective antibody response (Monteagudo et al., 2017). Currently, the molecular diagnosis of ASF is mostly based on real time PCR methods (qPCR), aimed at the detection of the conserved p72 gene (King et al., 2003; Zsak et al., 2005; Fernández-Pinero et al., 2013). These techniques have proved to be highly sensitive, but their main drawback is the requirement for specialized equipment and infrastructure, which implies the transport of samples to the diagnostic laboratory, prolonging the time to results. Hence, a rapid, pen-side molecular test, with a similar sensitivity to qPCR would be an exceptionally useful tool for ASF surveillance and control.

Loop-mediated isothermal amplification (LAMP) PCR methods have gained prominence as a feasible alternative for molecular diagnosis at the point of care (Knox and Beddoe, 2021; Lu et al., 2021; Sakthivel et al., 2021). These assays are based on the amplification of nucleic acid, using strand displacement polymerases, which can be carried out without the sudden temperature changes needed for conventional PCR (Notomi et al., 2000). This amplification can be visualized either by traditional electrophoresis methods, by detection of fluorescence, turbidity or color changes in the reaction (Mori et al., 2001; Parida et al., 2008; Tanner et al., 2015). The need for a minimum of 6 primer sequences within a ≈ 300 nt span for LAMP amplification, complicates the assay design, in terms of sensitivity, but also provides increased specificity.

Against this background, the present work focused on the development of a LAMP assay for the detection of ASFV. The sensitivity and specificity of the assay were evaluated against the molecular diagnostic techniques recommended by the USDA and the WOAH. The assay was also tested in a variety of matrices, aiming to provide a tool with potential to be used in field conditions. All the validation testing performed with the plasmid target was carried out at the veterinary diagnostic laboratory, University of Illinois at Urbana-Champaign, IL, USA (VDL-UIUC). The validation of the assays, using ASFV viral strains, was performed in the biosafety level 3 facilities at IRTA-CReSA, Barcelona, Spain.

## 2 Materials and methods

### 2.1 Cells and viruses

Collection of porcine alveolar macrophages (PAMs) from healthy donor pigs was carried out by bronchoalveolar lung lavages with PBS. Cells were cultured in RPMI 1640 medium, supplemented with 10% fetal bovine serum.

The Badajoz 71 virulent strain (BA71, Genotype I), isolated from the 1971 Spanish ASFV outbreak, was provided by Dr. ML Salas (CBMSO-CSIC, Madrid-Spain). The Georgia 2007/1 virulent strain (genotype II) was provided by Dr. Linda Dixon (The Pirbright Institute, Ash Road, Pirbright, Surrey GU24 0NF, UK).

Viral strains were titrated in PAMs by endpoint dilution, using a peroxidase‐linked assay. The virus titers were expressed as tissue culture infectious dose 50 (TCID_50_) per milliliter and calculated using standard statistical methods (Reed and Muench, 1938).

### 2.2 ASFV sequence analysis

The ASFV p72 gene was selected as the target for ASFV-LAMP primer design. All the full length (n=111) and partial (n=1304) p72 sequences available in NCBI were aligned using the BioEdit sequence alignment Editor, version 7.0.5.3 (Hall, 1999) and a consensus sequence was generated (1941 nt, >99% homology with field ASFV strains). The ASFV sequences alignment was then subjected to entropy analysis, to find the most conserved regions within the gene sequence. Selected gene regions that contained multiple conserved sequences (>15 continuous nts) within a 200 nt span were stored for further analysis.

### 2.3 Plasmid insert designs

The consensus sequence from ASFV p72 (1941 nt) was submitted to Integrated DNA technologies Inc. (IDT, Coralville, IA, USA) for custom gene synthesis and cloning into a plasmid vector expressing a kanamycin resistance gene (pUCIDT (Kan)). The pUCIDT (Kan) plasmid, with the p72 insert (named pSLA) was transfected by heat shock into competent E. coli (NEB^®^ DH5-alpha Competent *E. coli* (High Efficiency), New England Biolabs, Ipswich, MA) for propagation. After plasmid purification with the QIAGEN plasmid Midi Kit (Qiagen, Hilden, Germany), the p72 sequence insert was verified by Sanger sequencing. Plasmid quantification was carried out using the Qubit™4 Fluorometer system (Thermo Fisher Scientific, Waltham, MA) and plasmid ten-fold dilutions were performed, starting at a concentration of 10^7^ plasmid copies/µL until 1 plasmid copy/µL. The plasmid dilutions were used as standards and positive control for the ASFV-LAMP validation.

### 2.4 ASFV-LAMP primer design

The LAMP designer, version 1.16 software (Premier Biosoft, Palo Alto, CA, USA) was used for the design of the LAMP primers, based on the full-length consensus p72 sequence (1941 nts). The sequences from each primer in every primer set generated by the software, were evaluated by pairing them against the ASFV p72 full sequence alignment. Degenerations were manually introduced into each primer, in cases when the nucleotide (nt) showed <90% homology in all the analyzed sequences. Primer sets that included any “N” degenerations were discarded, as were those that included primers with >10% of degenerated nts in the primer sequence. Additionally, the OligoAnalyzer tool of IDT (https://www.idtdna.com/pages/tools/oligoanalyzer) was used to assess the complementarity in the primers. Both the non-degenerated, and the degenerated primer sequences were compared to check for major increases in complementarity associated with degenerations.

### 2.5 qPCR reference methods for ASFV detection

All viral culture and infected animal samples were subjected to viral DNA extraction, using the IndiMag Pathogen Kit (Indical, Leipzig, Germany) according to the manufacturer’s instructions. For all samples, an initial volume of 200 μl of sample were used for a final yield of 100 μl of DNA. Tissue samples were suspended in RPMI 1640 media (Lonza bioscience, Morrisville, NC) and macerated through a 70μm filter before DNA extraction. As an alternative to DNA extraction, a heating method for DNA release with minimal equipment was employed, when specified. The samples, diluted at a 1:10 or 1:100 ratio in sterile water, were then subjected to a heating at 94° C for 10 min before either qPCR or LAMP amplification.

Detection of ASFV DNA was carried out by the WOAH-recommended ASFV qPCR assay (Fernández-Pinero et al., 2013), using the modified protocol that employs the ASF-VP72P1 probe instead of the UPL probe, in accordance with the WOAH guidelines (World organisation for animal health, 2019b). This test is routinely used at IRTA-CReSA for ASFV surveillance, following the standard operating procedures of the European Union Reference laboratory for ASF at CISA-INIA (CISA-INIA, 2021). Cycle threshold (Ct) values below 35 were considered as positive, between 35 and 40 as suspect and above 40 as negative. Additionally, the USDA-validated ASFV qPCR assay (Zsak et al., 2005), routinely used in the VDL-UIUC for ASFV surveillance, was used for detection of pSLA DNA, with Ct values under 40 being considered positive.

### 2.6 Fluorometric LAMP reactions

Fluorometric LAMP reactions were performed in a final reaction volume of 25µL under the following conditions: Fluorometric LAMP Mastermix (ISO-004, OptiGene Limited, West Sussex, UK) 1X, FIP and BIP primers (0.8µM), LoopF and LoopB primers (0.4µM), F3 and B3 primers (0.2µM) and DNA template (5µL). LAMP amplification was carried out at 65°C for 30 min, in the Genie^®^ II LAMP machine (OptiGene), unless indicated otherwise.

The annealing temperature of the pSLA amplifications was used to establish the annealing temperature for ASFV-LAMP reactions. Samples were considered as positive if there was a detectable amplification peak before the end of the amplification time, as well as an annealing temperature within 0.5°C of that established for the positive controls.

### 2.7 Colorimetric LAMP reactions

Colorimetric ASFV-LAMP reactions were performed under the following conditions: WarmStart^®^ Colorimetric LAMP Master Mix (New England biolabs, Ipswitch, MA) 1X, FIP and BIP primers (0.8µM), LoopF and LoopB primers (0.4µM) and F3 and B3 primers (0.2µM). LAMP amplification was carried out at 65°C, either in a thermocycler (GeneAmp^®^ PCR system 2700, Applied biosystems) or in a water bath, using pSLA DNA as template (5µL/reaction). Final reaction volume was set at 25µL. Amplification was carried out at 65°C for 30 minutes. Reactions were deemed as positive (bright yellow), suspect (pale pink) or negative (bright pink), according to the coloration of the mastermix at the end of the amplification.

### 2.8 Thermal stability

The thermal stability of the ASFV-LAMP amplification was tested for both fluorometric and colorimetric reactions, in a temperature gradient between 60-70°C, using the temperature gradient function of the Genie^®^ II LAMP machine. The LAMP reactions were performed using the pSLA DNA at a concentration of 10^4^ plasmid copies/µL.

### 2.9 LAMP analytical limit of detection

Ten-fold dilutions of pSLA DNA were used to assess the analytical limit of detection for the ASFV-LAMP assays, starting at a concentration of 10^6^ plasmid copies/µL through 1 plasmid copy/µL. The dilutions were tested in triplicate by both fluorometric and colorimetric LAMP and paired against the Ct value obtained by the USDA-validated ASFV qPCR assay (Zsak et al., 2005).

### 2.10 LAMP biological limit of detection

The ASFV BA71 and Georgia 2007/01 strains, cultured in PAMs, were used to evaluate the biological limit of detection of the LAMP assays. Ten-fold dilutions of ASFV-infected PAMs were performed, starting at a titer of 10^5^ TCID_50_/ml and 10^7.2^ TCID_50_/ml for BA71 and Georgia 2007/01, respectively. Each viral dilution was subjected to column-based DNA extraction and viral DNA was stored at -80°C. Samples were tested, in triplicate, by fluorometric and colorimetric LAMP and compared to their respective Ct value by the WOAH-recommended ASFV qPCR (Fernández-Pinero et al., 2013).

### 2.11 LAMP testing of DNA-spiked samples

In order to assess the inhibitory capabilities of different matrices on the ASFV-LAMP assays, four different matrices were tested: Sera, Tonsil swabs, rectal swabs and oral fluids. Swab samples had been diluted in 500 µl of water. Ten samples from each of the four matrices were diluted in water at a 1:10 proportion and afterwards were spiked with pSLA DNA at a concentration of 5*10^2^ plasmid copies/uL. Additionally, to simulate a nucleic acid extraction method to be easily performed in field conditions, the samples were boiled at 100°C for 15 minutes (Ceruti et al., 2021; Yang et al., 2022). Afterwards, non-spiked and spiked samples were evaluated by fluorometric and colorimetric LAMP detection. Half of the assays were performed by one operator and the other half by another, to assess the possible effect of the operator on the results.

### 2.12 Detection of ASFV DNA from experimentally infected animals

Samples obtained from animals experimentally infected with either ASFV genotype I or II (n=26 samples for each genotype) were randomly selected and evaluated in duplicates using ASFV-LAMP. These samples were also tested by the WOAH-recommended qPCR test and results were compared to those obtained with the ASFV-LAMP.

For genotype I, samples corresponded with a previously performed experimental infection in wild boar, using the wild type E75 strain (Cabezón et al., 2017), were tested. Sample matrices included: nasal swab, rectal swab, lymph node, bone marrow, lung, tonsil, spleen, and sera. In the case of genotype II, domestic pigs had been infected with the Georgia2007/01 strain at a dose of 10^4^ hemadsorption units (HA_50_)/ml, through intranasal inoculation. Sample matrices included: Nasal swab, oral swab, rectal swab, lymph node, lung, tonsil, sera, and whole blood. The inoculation took place at the biosafety level 3 facilities (BSL3) at IRTA-CReSA and was carried out according to existing Spanish and European regulations. The protocol had been approved by the Ethical Committee of the Generalitat de Catalonia, Spain under the animal experimentation project number 11768.

### 2.13 ASFV-LAMP detection of viral DNA extracted using minimal equipment

Samples from the ASFV experimentally infected animals (section 2.12) were randomly selected and diluted at a 1:10 or 1:100 ratio in sterile water, followed by either magnetic bead-based or heating-based extraction, as described in section 2.5. The DNA obtained from both extraction methods was tested by the WOAH-qPCR and by ASFV-LAMP. A total of 12 samples (six from genotype I and six from genotype II) were used. Randomized selection led to three overlapping matrices that were analyzed for both genotypes (serum, rectal swab and lymph node). Lung, tonsil and spleen samples were only tested in genotype I-infected animals, whereas whole blood, oral swab and bone marrow were only tested from genotype II-infected pigs.

### 2.14 Specificity testing

Primer sets ASFV-LAMP-BG2 and ASFV-LAMP-BG3 were tested against a battery of DNA samples from 22 different pathogens (**Supplementary Table 1**). These samples corresponded with bacterial and viral pathogens relevant for porcine health, as well as pathogens genomically related to those of relevance in porcine health.

### 2.15 Statistical analysis

Statistical analysis was performed using the GraphPad Prism 8.3.0 software for Windows (GraphPad Software, San Diego, California USA, www.graphpad.com). The Mann-Whitney non-parametric test was used for comparisons of means between groups.

## 3 Results

### 3.1 Selection of ASFV-LAMP primer candidates

**Figure 1:**
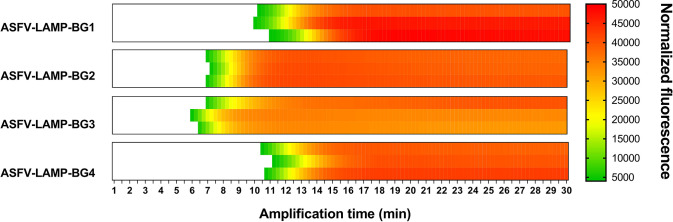
Comparison of the fluorometric ASFV-LAMP PCR amplification by the four designed primer sets. Amplification is represented as the normalized fluorescence, shown in a colorimetric scale, of the three replicates performed using each primer set.

Using the established criteria, four primer sets (named ASFV-LAMP-BG1 to ASFV-LAMP-BG4) were selected for testing. The fluorometric LAMP results showed detection of pSLA DNA by all the synthesized primer sets (**Figure 1**). The fastest ASFV DNA amplification was detected for primer set ASFV-LAMP-BG3 which had detectable fluorescence between 5-6 minutes. A slight increase in the time to detection was found for the ASFV-LAMP-BG2, which showed positive results starting at 7 minutes, whereas the amplification time was increased by over 4 minutes in the remaining two primer sets (**Figure 1**). None of the primer sets tested showed non-specific amplification detectable in the negative controls.

Due to its shorter time to detection, primer sets ASFV-LAMP-BG2 and ASFV-LAMP-BG3 were selected for further validation. The primer sequences and their location within the ASFV-p72 gene are detailed in **Table 1**.

**Table 1:** Primer sequences for the ASFV-LAMP-BG2 and ASFV-LAMP-BG3 primer sets.

Primer set	Primer	Sequence	Position
ASFV-LAMP-BG2	F3	CAAGATCAGCCGTRGTGATAG	72-92
	***B3***	***TCCGTAACTGCTCATGGTA***	***321-300***
	FIP(***F1c*** +F2)	***CCTTTGCTTTGAAGCCACGG*** AATTCTCTTGCTCTGGATACG	***194-175***; 125-145
	BIP(B1c+ ***B2***)	CATCATCGCVCCCGGATCRT ***AGTTCTGCAGCTCTTACATAC***	199-218; ***275-255***
	***LoopF***	***GAGGAATACCAACCCAGTGG***	***174-155***
	LoopB	ATYGMATTGCCTCCGTAG	230-247
ASFV-LAMP-BG3	F3	GTTGCGTCCGTRATAGGRG	1037-1055
	***B3***	***ATGACTGGATATAAGCACTTGG***	***1281-1260***
	FIP(***F1c*** +F2)	***CGAACGTGYAGCCATACCA*** GGATATTGTGVGAGTTCTCGG	***1146-1128***; 1080-1098
	BIP(B1c+ ***B2***)	GCTTTGGTGCGGCTTGTG ***CAGGAGGTATCGGTGGAG***	1180-1197; ***1254-1238***
	***LoopF***	***CCCGAAATTYCTTTCACARCAT***	***1126-1105***
	LoopB	TGAATGTTGCATAGGAGAGGG	1205-1225

**In bold-Italics**: sequences in reverse-complementary form.

### 3.2 The ASFV-LAMP-BG2 and ASFV-LAMP-BG3 primer sets showed thermal stability

**Figure 2:**
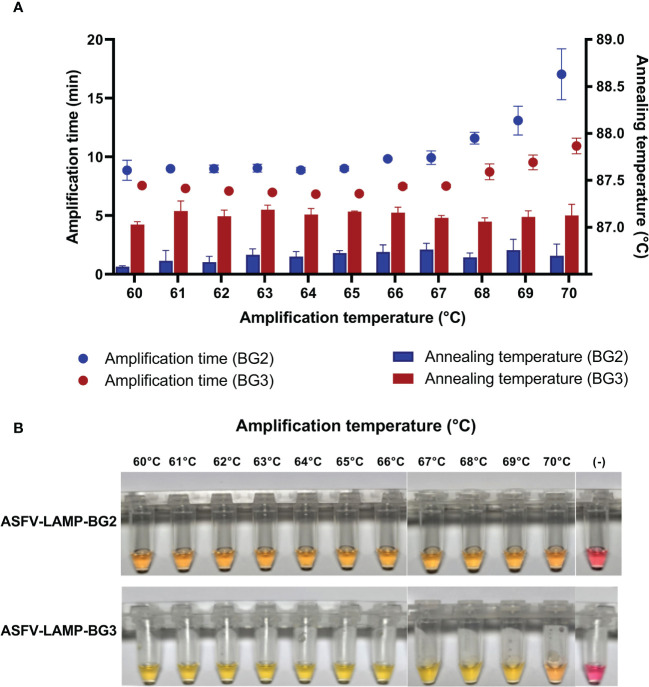
Thermal stability of ASFV-LAMP. **(A)** Fluorometric detection of ASFV-LAMP-BG2 (blue dots and bars) and ASFV-LAMP-BG3 (red dots and bars) amplification. Dots represent the time to detection (in minutes, left Y axis) and bars represent the annealing temperature for each reaction (in °C, right Y axis). **(B)** Colorimetric detection of ASFV-LAMP-BG2 (upper panel) and ASFV-LAMP-BG3 (lower panel) showing amplification at different temperatures. Reactions were deemed as positive when a discernible color change could be observed in the mastermix, compared with the negative control (far right tube).

In the fluorometric assay, the pSLA DNA (10^4^ plasmid copies/µL) was amplified by both ASFV-LAMP-BG2 and ASFV-LAMP-BG3 primer sets at all the amplification temperatures tested (between 60 and 70°C, **Figure 2A**). The shortest times to detection were found in the reactions carried out at temperatures <67°C for both primer sets. Annealing temperatures showed small variations between the tested temperatures and were found to be between 86.5 ± 0.5°C for ASFV-LAMP-BG2 and between 87.0 ± 0.5°C for ASFV-LAMP-BG3 (**Figure 2A**).

Both ASFV-LAMP assays showed positive results, using colorimetric detection, after 30 min of amplification at all the temperatures between 60-68°C (**Figure 2B**). The mastermix coloration was clearly differentiated between the positive samples (yellow) and the negative controls (bright pink).

### 3.3 The two selected ASFV-LAMP assays are able to detect as few as 1 gene copy/µL

**Figure 3:**
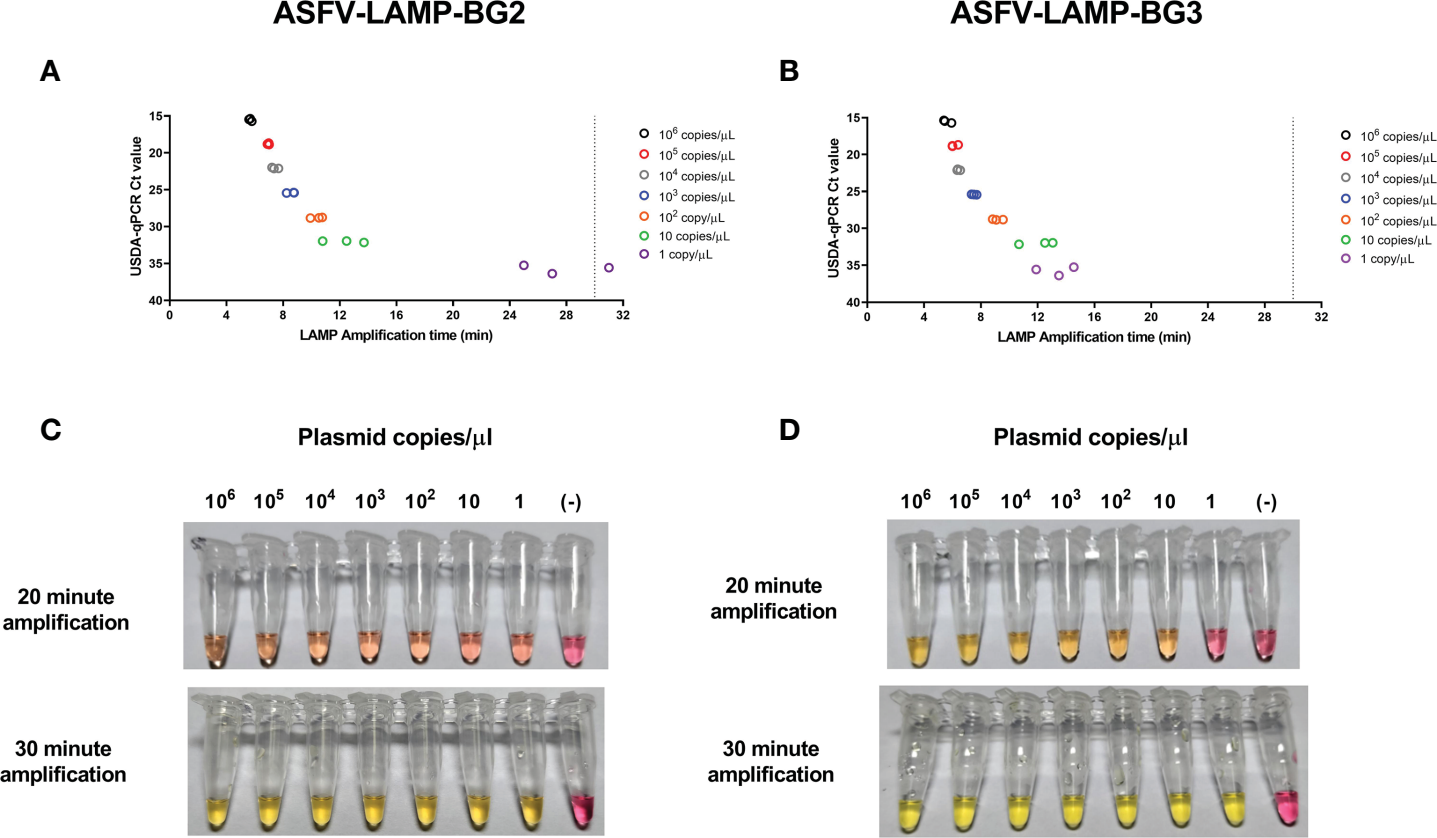
Analytical limit of detection for the ASFV-LAMP-BG2 and ASFV-LAMP-BG3 assays. Ten-fold dilutions of the pSLA plasmid (containing the ASFV-p72 gene sequence) were tested by fluorometric **(A, B)** and colorimetric **(C, D)** LAMP, as well as by the USDA-reference qPCR test. For the fluorometric detection, results are represented as the time to detection (in minutes, X axis), compared to the qPCR results, expressed in Ct value (Y axis). Samples that showed LAMP amplification before 30 minutes (dotted line), were considered as positive. Colorimetric reactions were checked after 20 and 30 minutes of amplification and were deemed as positive when a discernible color change could be observed in the mastermix, compared with the negative control (far right tube).

Using fluorometric detection, amplification was detected as early as 5 minutes in the highest pSLA concentration tested (10^6^ plasmid copies/µL, Ct value ≈ 16 by qPCR), by either ASFV-LAMP-BG2 or ASFV-LAMP-BG3 (**Figures 3A, B**). Both ASFV-LAMP assays were capable to detect all the subsequent ten-fold dilutions, showing similar amplification times up to 10 plasmid copies/µL. In the lowest plasmid concentration tested (1 copy/µL), the ASFV-LAMP-BG3 primer set showed better performance, detecting all the triplicates tested, with an amplification time ≈ 13 minutes, whereas the ASFV-LAMP-BG2 failed to detect one of the triplicates and showed amplification time over 20 minutes. The Ct value from this plasmid dilution by qPCR was around 36, near the limit of detection of the technique.

For the colorimetric assay, color changes in the mastermix from both ASFV-LAMP-BG2 and ASFV-LAMP-BG3 assays were observed as early as 20 min of amplification, particularly at the highest plasmid concentrations (**Figures 3C, D**). Primer set ASFV-LAMP-BG3 showed a more noticeable yellow coloration at this time-point. Following an extra 10 min amplification, all the reactions with ASFV plasmid dilutions showed a bright yellow color, in contrast with the bright pink of the negative control (**Figures 3C, D**). For the above reasons, primer set BG3 became the primary target of further testing.

The ASFV-LAMP-BG3 assay also proved to be highly specific, since none of the nucleic acid samples from other pathogens was positive by fluorometric nor colorimetric LAMP (**Supplementary Table 1**).

### 3.4 The ASFV-LAMP-BG3 assay detected target DNA in a variety of matrices

**Figure 4:**
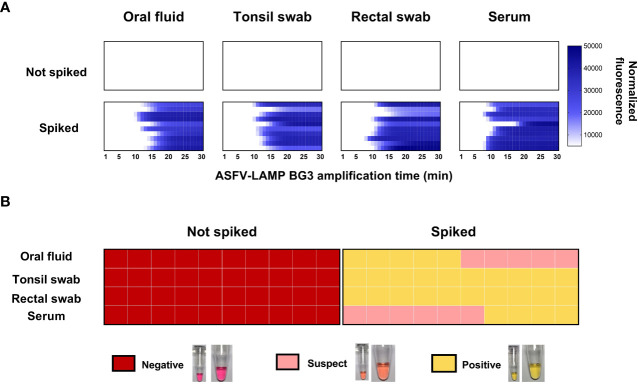
Detection of pSLA DNA (containing the ASFV-p72 gene sequence) in spiked matrices by ASFV-LAMP-BG3. **(A)** Fluorometric detection of pSLA DNA. Amplification is represented as the normalized fluorescence, shown in a colorimetric scale, of the spiked samples. **(B)** Colorimetric detection of ASFV-LAMP-BG3 amplification. Reactions were characterized as positive, suspect or negative, according to the coloration of the mastermix after 30 min of amplification.

None of the matrices tested showed non-specific amplification, neither in the fluorometric, nor the colorimetric assays. Conversely, all the spiked samples were positive by fluorometric detection. Amplification times in the fluorometric LAMP detection were similar for all the matrices tested, as well as for both operators, with no statistical differences being evidenced (**Figure 4A**).

In the colorimetric detection, the ASFV-LAMP-BG3 primer set showed clearly positive results in over 70% of pSLA-spiked samples, while the remaining samples showed pale pink coloration, corresponding with suspect results. Nevertheless, the coloration on the “suspect” samples was still clearly differentiated from the negative controls and the unspiked samples (**Figure 4B**).

### 3.5 The ASFV-LAMP assays showed highly efficient detection of ASFV DNA from multiple viral genotypes

**Figure 5:**
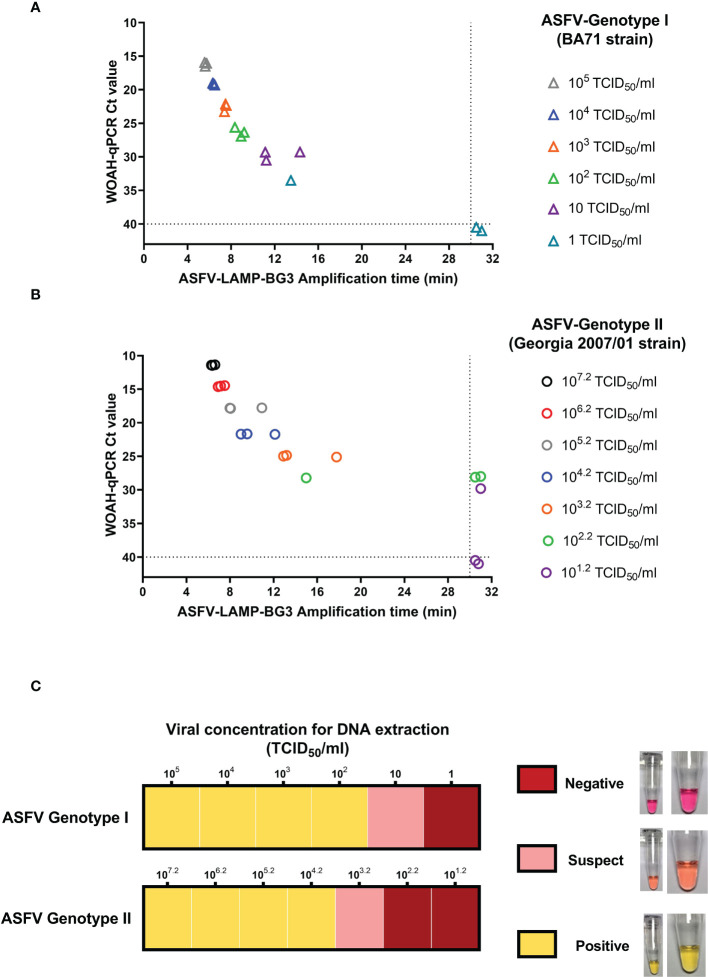
Detection of ASFV genotypes I and II by LAMP PCR. Ten-fold dilutions of viral cultures from ASFV Badajoz 71 strain (genotype I, panels **A** and upper panel of **C**) and Georgia 2007/01 strain (genotype II, panel **B** and lower panel of **C**) were tested by the ASFV-LAMP-BG3 primer set. Results of fluorometric LAMP are represented as the time to detection in minutes (panels **A** and **B**, X axis) in comparison with the Ct value from the WOAH recommended qPCR assay (panels **A** and **B**, Y axis). Samples that showed LAMP amplification before 30 minutes (dotted line), were considered as positive. Colorimetric results are shown in a qualitative scale, in accordance with the mastermix coloration after 30 min amplification.

In the ASFV genotype I strain (BA71), amplification time for the highest DNA concentration tested (10^5^ TCID_50_/ml, Ct value ≈ 16 by qPCR) was around 5:30 minutes by ASFV-LAMP-BG3, increasing by about one minute for each subsequent dilution. The ASFV-LAMP assay was able to consistently detect ASFV DNA until the sample extracted from the 10 TCID_50_/ml virus dilution, while one of the triplicates from the 1 TCID_50_/ml dilution was also positive (**Figure 5A**). Replicates that were negative by ASFV-LAMP assays were also negative by the WOAH-recommended ASFV qPCR test. Using colorimetric detection, ASFV-LAMP-BG3 showed bright yellow coloration in the majority of the genotype I viral DNA samples, corresponding with clear positive results. Only the lowest concentration detected was characterized as suspect (pale pink coloration).

Amplification times of ASFV genotype II DNA (Georgia2007/01 strain) ranged from 6 to 17 minutes in samples with titers between 10^7.2^ and 10^2.2^ TCID_50_/ml, using fluorometric detection for ASFV-LAMP-BG3. The LAMP assay was able to consistently detect DNA from viral dilutions as low as 10^3.2^ TCID_50_/ml (Ct values of approximately 28) and showed partial detection at lower concentrations, near the limit of detection for the WOAH-recommended qPCR assay (**Figure 5B**). In the colorimetric LAMP tests, the lowest DNA concentration detected corresponded with viral dilution with a titer of 10^3.2^ TCID_50_/ml. This was also the only DNA concentration showing a suspect result, with the rest being clearly positive (**Figure 5C**).

### 3.6 The ASFV-LAMP-BG3 primer set was highly efficient at detecting viral DNA from ASFV-infected tissues and clinical samples

**Figure 6:**
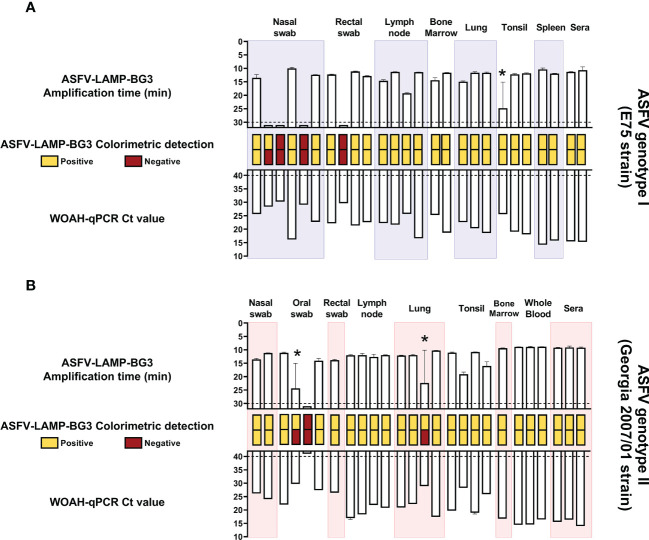
ASFV-LAMP-BG3 testing in samples from infected animals. Samples from wild boar infected with the Badajoz 71 strain (genotype I, panel **A**) or pigs infected with the Georgia2007/01 strain (genotype II, panel **B**) were tested by ASFV-LAMP in duplicate, using fluorometric and colorimetric detection, as well as by the WOAH-recommended qPCR. Fluorometric LAMP results are represented as time to amplification with each bar representing one sample (upward facing bars) and error lines showing the variability in the LAMP duplicates. Samples that showed LAMP amplification before 30 minutes (dotted line), were considered as positive. Asterisk represents samples with one positive and one negative replicate by LAMP-PCR. Colorimetric LAMP results are shown in a qualitative scale, with duplicates represented as two small, stacked rectangles between the LAMP and qPCR bars. qPCR results are shown as Ct value (downward facing bars). Samples that showed a Ct value below 40 were considered as positive.

All the sera and tissue samples evaluated from E75-infected wild boar (ASFV genotype I) were positive by both fluorometric and colorimetric detection using the ASFV-LAMP-BG3 primer set, though one of the replicates from a tonsil sample was negative by fluorometric LAMP (**Figure 6A**). Half of the nasal swab samples evaluated were negative by fluorometric LAMP detection, as was one of the rectal swab samples. These samples corresponded with the lowest DNA concentrations tested, showing CT values >28 by the WOAH-recommended qPCR.

In the case of Georgia2007/01-infected animals (ASFV genotype II), all the samples that were positive by the WOAH-recommended qPCR test were also positive in at least one replicate, by both fluorometric and colorimetric LAMP (**Figure 6B**). The two samples with one positive and one negative replicate in both detection techniques (one lung and one oral swab), had also shown the highest CT values by qPCR. The annealing temperature for all the samples from animals infected whether with genotype I or II ASFV, was within 1° C of variability (86.08 – 87.07).

### 3.7 ASFV-LAMP amplification can be efficiently performed using minimal equipment DNA extraction

**Figure 7:**
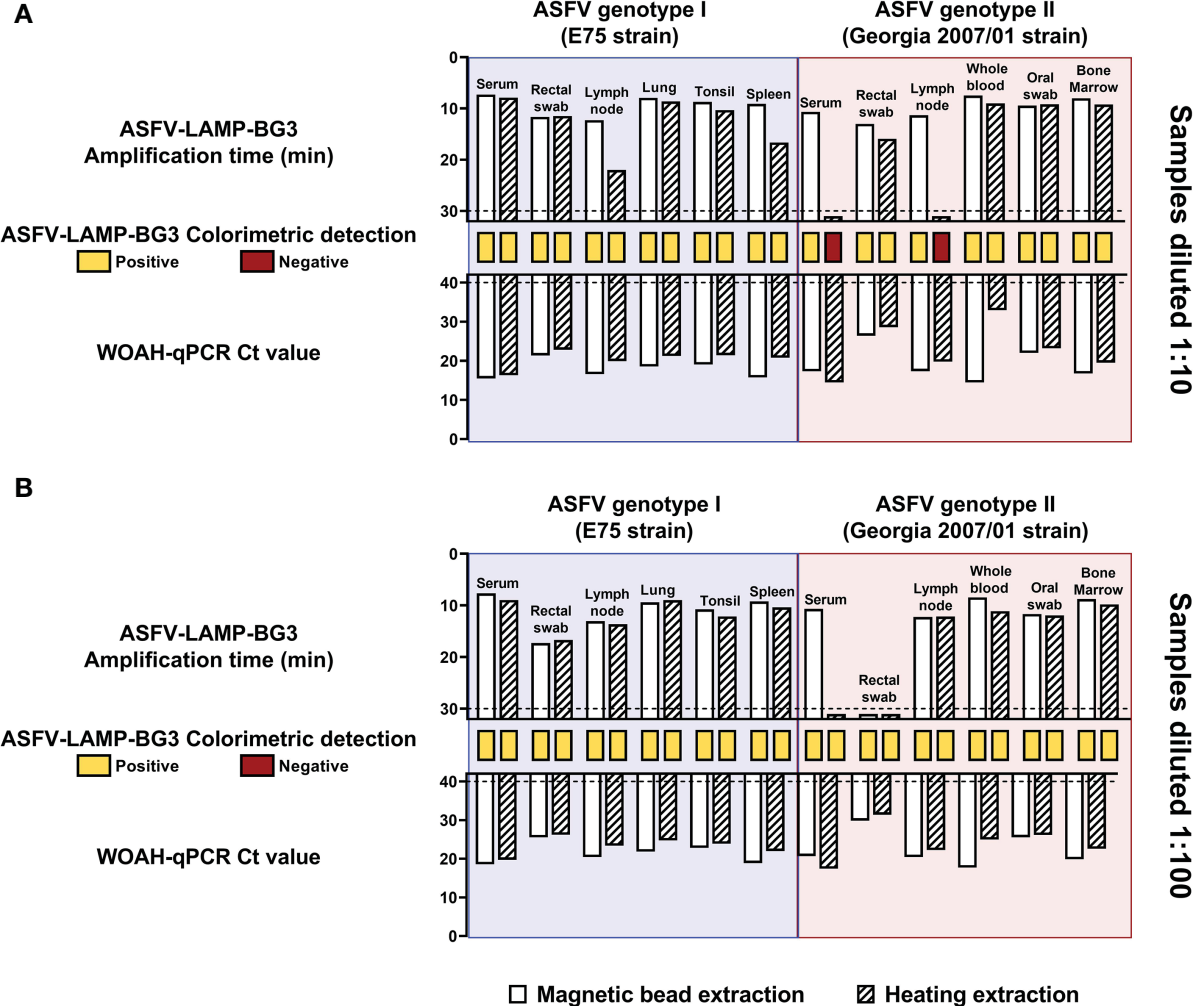
Evaluation of ASFV-LAMP-BG3 using an equipment-free DNA extraction method. Samples from animals infected with ASFV were diluted at a ratio of 1:10 **(A)** or 1:100 **(B)** and subjected to magnetic bead-based (plain bars) or equipment-free extraction based on heating (striped bars). DNA samples were tested by ASFV-LAMP, using fluorometric and colorimetric detection, as well as by the WOAH-recommended qPCR. Animals had been infected either with the Badajoz 71 strain (genotype I, blue shaded panels) or the Georgia2007/01 strain (genotype II, red shaded panels). Fluorometric LAMP results are represented as time to amplification with each bar representing one sample (upward facing bars). Samples that showed LAMP amplification before 30 minutes (dotted line), were considered as positive. Colorimetric LAMP results are shown in a qualitative scale, as rectangles between the LAMP and qPCR bars. qPCR results are shown as Ct value (downward facing bars). Samples that showed a Ct value below 40 were considered as positive.

In the samples diluted at a 1:10 ratio, animals that were infected with genotype I ASFV were positive in the six matrices tested by both ASFV-LAMP-BG3 and qPCR, regardless of the extraction method used. Ct values and amplification time were slightly higher in the DNA samples extracted through heating treatment (**Figure 7A** blue). Colorimetric LAMP detection was also positive in both the magnetic bead-based and the heating-based DNA extraction from these samples. In the case of genotype II-infected pigs, the serum and lymph node samples that had been subjected to heating extraction were not amplified by ASFV-LAMP, neither by fluorometric nor colorimetric detection, despite being positive by qPCR. The remaining samples from animals infected with genotype II ASFV were positive by qPCR and both ASFV-LAMP detection methods (**Figure 7A** pink)

For the samples diluted at a proportion of 1:100, all the samples from animals infected with ASFV genotypes I or II were positive by qPCR and colorimetric LAMP detection, using both DNA extraction methods (**Figure 7B**). In the ASFV-LAMP fluorometric detection, only the heat-extracted serum sample and the rectal swab sample were negative (**Figure 7B**).

## 4 Discussion

The current epidemiological situation of ASFV continues to pose a great concern for the swine industry worldwide (Sauter-Louis et al., 2021a; Sauter-Louis et al., 2021b; Ito et al., 2022). This is exacerbated by the lack of a global vaccination strategy against the virus, which renders surveillance as the basis around which prevention and control strategies are built (Costard et al., 2009; Urbano and Ferreira, 2022). The currently used techniques, while being highly sensitive and specific, entail a delay of the time to obtain a result, given the requirement for special equipment and infrastructure to carry them out (World organisation for animal health, 2019b). The results of the present study show the design of an ASFV-LAMP PCR primer set, which can be employed for the detection of ASFV DNA using portable equipment, potentially reducing the time to diagnosis. Additionally, this primer set (ASFV-LAMP-BG3) proved to perform efficiently with a colorimetric detection scheme requiring minimal equipment, making it adaptable to point-of-care testing.

The ASFV-LAMP primer sets were designed based on the ASFV-p72 gene, which shows highly conserved regions across different genotypes. This gene has proven to be very reliable as a target for ASFV molecular diagnosis and is currently used for the WOAH and USDA recommended qPCR assays (King et al., 2003; Zsak et al., 2005; Fernández-Pinero et al., 2013). Using this target, the ASFV-LAMP assay proved to reliably detect as few as 1 target copy/µl (5 target copies/reaction), while being highly specific and not showing amplification of any other tested microorganisms. This analytical limit of detection proved to be similar to that of the WOAH-recommended qPCR assay (Fernández-Pinero et al., 2013). Remarkably, the highly sensitive detection of ASFV DNA by ASFV-LAMP-BG3 was achieved using both fluorometric and colorimetric detection methods. This primer set consistently showed a clear differentiation between negative and positive samples, in the colorimetric detection assays, particularly those carried out with viral DNA.

Recently, the WOAH identified point of care isothermal techniques, such as LAMP, as promising technologies for ASFV field diagnosis, but showed that the relatively high cost of detection equipment remains their main drawback (Inui et al., 2022). The results of the present study directly address these concerns with the development of an ASFV-LAMP PCR assay that can be performed with minimal equipment (i.e. a water bath). Another concern for the implementation of these techniques in the field relates to the need for DNA extraction, which also requires equipment that is not readily available in the field (Inui et al., 2022). In this regard, previous studies have shown that pathogen lysis through heating, can be used for nucleic acid release without the need for extraction columns or laboratory equipment (Ceruti et al., 2021; Yang et al., 2022). However, these studies have been performed using reagents, such as lysis buffers and pH stabilizers, that are not readily available at the point of care. In the present study, this DNA release method was applied to tissues from animals infected with ASFV, using water as a diluent, instead of a commercial dilution lysis buffer for the samples before DNA release through heating. The fact that all these samples were positive by qPCR after heating extraction shows that DNA release using water was successful. This was replicated by boiling the DNA-spiked samples for 15 minutes, which yielded positive LAMP-PCR results. Nevertheless, in the tissues from animals infected with ASFV, not all of the matrices tested showed a positive result. It is possible that certain matrices can hinder LAMP amplification, or the detection methods used, and thus require further dilution. This appeared to be the case in the present study, in which sample dilution at a proportion of 1:100 yielded 100% positive results in the colorimetric detection. This is noteworthy, considering that this is the cheaper and most feasible method to be implemented as a pen-side test, due to the lack of equipment that would be needed. Therefore, the application of this method would be of great relevance for the active surveillance of the virus, and it can be used in animal movement transport, on farms and even in areas where dead boar carcasses can be detected.

Considering the potential advantages posed by isothermal amplification techniques for the development of point of care diagnostic tests, multiple isothermal assays have recently been developed for the detection of ASFV (Mee et al., 2020; Ceruti et al., 2021; Wang et al., 2021a; Yang et al., 2022). However, their detection methods rely on specialized equipment or dyes (such as SYBR green). Lateral flow approaches, associated with some of these techniques have also been reported to be subject to cross contamination risk (Ceruti et al., 2021). Comparatively, the ASFV-LAMP assay designed in the present study provides either faster detection or requires minimal equipment, while continuing to show high performance through fluorometric and colorimetric detection. Additionally, the interest spiked by LAMP technologies has led to recent improvements in terms of development of thermostable enzymes (facilitating travel), as well as portable detection methods, using smartphones, that can potentially be applied to the designed ASFV-LAMP-BG3 assay (Chander et al., 2014; Jankelow et al., 2022).

Moreover, the matrices used for spiking in the present study corresponded with those generally received at the VDL and at IRTA-CReSA for surveillance of ASFV or other pathogens. These included samples that are routinely taken by veterinarians in the field or at slaughterhouses (serum and tonsils), as well as some obtained through minimally invasive methods that do not require a high level of training to collect in the field (oral fluids and rectal swabs). The ASFV-LAMP tests showed high performance in the great majority of matrices tested, suggesting that this strategy could be applied in field conditions with minimal training.

It should be noted that the ASFV-LAMP PCR assay developed for the present study (using fluorometric and colorimetric detection) was designed with the aim to be useful for detection of as many ASFV genotypes as possible and proved to detect viral DNA from genotypes I and II. This is particularly important, considering that nearly all ASFV outbreaks since the reintroduction of the virus in Europe, Asia and the Caribbean have been related to genotype II strains (Ge et al., 2018; Gallardo et al., 2021; Mighell and Ward, 2021). Additionally, genotype I has recently been found to be reemerging in China (Sun et al., 2021), making both of these genotypes highly important targets for surveillance in countries with large pork production. Notably, even though the ASFV-LAMP assays showed a similar analytical limit of detection to the reference qPCR methods, this seemed to be higher when testing viral DNA rather than plasmid DNA. This could be due to mutations in individual viral particles that may hinder primer hybridization, which would only affect amplification at lower DNA concentrations. Another possible explanation would be that the DNA extracted from plasmids, besides being more uniform, also had higher integrity after extraction. It has been shown that DNA integrity, influenced by the extraction method used, is a factor that may affect LAMP PCR results (Ablordey et al., 2021). This is in line with testing performed in our lab which showed that freezing-thawing of DNA samples increased the amplification time by LAMP (Data not shown).

In any case, the lowest viral DNA concentration detected by the ASFV-LAMP assays (10 and 10^2.2^ TCID_50_/ml, for genotypes I and II, respectively) was well within the values observed during ASFV experimental infections (Cabezón et al., 2017; Lohse et al., 2022). However, it also indicates that the efficiency of the LAMP tests, while very good, remains inferior to the reference qPCR tests, and should therefore be considered as complementary to these techniques. In addition, the use of ASFV-LAMP testing should likely be limited to samples in which high viral DNA load is likely to be found, considering that samples corresponding with the highest qPCR Ct values, such as nasal swabs, showed the poorest performance in ASFV-LAMP.

The capability of the ASFV-LAMP- BG3 tests to detect some of the less common ASFV genotypes, which are mostly circulating solely in parts of Africa (Mulumba-Mfumu et al., 2019; Njau et al., 2021; Qu et al., 2022), should be assessed in future studies. In this regard, the portability of a LAMP PCR machine for fluorometric detection or the austere setup with which the colorimetric LAMP can be performed, would suggest that these tools can be applied for surveillance even in remote regions or backyard farms. This is important, considering that, as high as 98% of the outbreaks have been related to backyard productions in the Dominican Republic (Gonzales et al., 2021; Rademacher et al., 2022). In addition, wildlife plays a key role in the epidemiology of ASFV and must be taken into account in any surveillance program (Mighell and Ward, 2021; Sauter-Louis et al., 2021a; Urbano and Ferreira, 2022). It should also be in the interest of swine producers worldwide to maintain surveillance of the disease in the regions where it had been confined, since this would likely prevent further outbreaks.

Taken together, the results from the present study provide two promising ASFV-LAMP tools (fluorometric and colorimetric) for the surveillance of ASFV, with the potential to be implemented in various field situations in a simple and practical way. The high analytical sensitivity and specificity of the ASFV-LAMP assays developed in this work suggest they might be highly valuable for their use in a laboratory setting, while the relative easiness with which they can be performed using minimal equipment means they can be implemented in field conditions. The potential impact of the significant decrease in time to detection afforded by these techniques, cannot be understated. The ASFV-LMAP-BG3 assay can be used as a primary screening test, to take preliminary action (such as temporary quarantine) before definitive diagnosis with a more standardized diagnostic technique. This could allow for faster implementation of the control protocols and therefore lead to a reduced impact of a potential outbreak, with severe implications in economic and animal welfare terms.

## Data availability statement

The original contributions presented in the study are included in the article/**Supplementary Materials**, further inquiries can be directed to the corresponding author/s.

## Ethics statement

The protocol had been approved by the Ethical Committee of the Generalitat de Catalonia, Spain under the animal experimentation project number 11768.

## Author contributions

Conceptualization, LG. Methodology, JA, SL, RR, MP-S, MA, FR, LG, and CM. Validation, JA, SL, RR, MP-S, MA, LG, and CM. Formal analysis, JA, LG, and CM. Resources, LG and CM. Data curation, JA, LG, and CM. Writing—original draft preparation, JA, SL, LG, and CM. Writing—review and editing, JA, SL, FR, LG, and CM. Visualization, JA. Supervision, LG and CM. Project administration, LG and CM. Funding acquisition, LG and CM. All authors contributed to the article and approved the submitted version.
