# Supplementary material for: Efficient detection of African Swine Fever Virus using minimal equipment through a LAMP PCR method
Source: Front Cell Infect Microbiol. 2023 Jan 27;13:1114772. doi: 10.3389/fcimb.2023.1114772 (PMC9911463; doi:10.3389/fcimb.2023.1114772)
Supplement: Supplementary file 1 [file Table_1.docx]

Supplementary Material

# Supplementary Table 1. Pathogens tested by the ASFV-LAMP-BG2 and ASFV-LAMP-BG3 primer sets

| **Sample ID** | **Pathogen** |
| --- | --- |
| CSFV | Classical swine fever virus |
| NA PRRSV | North American Porcine reproductive and respiratory syndrome |
| EU PRRSV | European Porcine reproductive and respiratory syndrome |
| SECD | Swine enteric coronavirus disease |
| SVA | Seneca valley virus |
| SIV | Swine influenza virus |
| PCV2 | Porcine circovirus 2 |
| PCV3 P5 | Porcine circovirus 3 |
| PCV3 P7 | Porcine circovirus 3 |
| PCV3 P3 | Porcine circovirus 3 |
| BVDV I | Bovine viral diarrhea I |
| BVDV II | Bovine viral diarrhea II |
| BCoV | Bovine Coronavirus |
| CHV | Canine herpesvirus |
| CPV2 | Canine parvovirus |
| APP | *Actinobacillus pleuropneumoniae* |
| *S. suis* | *Streptococcus suis* |
| *M hyoneumoniae* (lung) | *Mycoplasma hyopneumoniae* |
| *M hyoneumoniae* (organ pool)) | *Mycoplasma hyopneumoniae* |
| *M. hyorhinis* | *Mycoplasma hyorhinis* |
| CSFV plasmid | Classical swine fever virus 5' UTR |
| CDV | Canine distemper virus |
| CaDV | Canine adenovirus |
| *E. coli* | *Escherichia coli* |
| *G. parasuis* | *Glaesserella parasuis* |
| *E. rhusiopathiae* | *Erysipelothrix rhusiopathiae* |
